# Preoperative Management of MGD Alleviates the Aggravation of MGD and Dry Eye Induced by Cataract Surgery: A Prospective, Randomized Clinical Trial

**DOI:** 10.1155/2019/2737968

**Published:** 2019-04-11

**Authors:** Peng Song, Zhuo Sun, Shengwei Ren, Kaili Yang, Guohua Deng, Qingyan Zeng, Yajie Sun

**Affiliations:** ^1^Aier School of Ophthalmology, Central South University, Changsha, China; ^2^Hankou Aier Eye Hospital, Wuhan, China; ^3^Wuhan Aier Eye Institute, Wuhan, China; ^4^The Third People's Hospital of Changzhou, Changzhou, China; ^5^Provincial People's Hospital, Henan Eye Hospital, Henan Eye Institute, People's Hospital of Zhengzhou University, Zhengzhou, China

## Abstract

**Purpose:**

To investigate the effect of preoperative treatment and postoperative enhanced anti-inflammatory treatment on alleviating meibomian gland dysfunction (MGD) and dry eye induced by cataract surgery.

**Design:**

Prospective, randomized clinical trial.

**Methods:**

A total of 120 cataract patients with moderate obstructive-MGD were enrolled and randomized with 60:30:30 number of patients in cohorts I, II, and III, respectively: Cohort I: routine postoperative anti-inflammatory treatment; Cohort II: preoperative treatment (warming compress, lid hygiene, and anti-inflammatory treatment) and routine postoperative anti-inflammatory treatment; Cohort III: enhanced postoperative anti-inflammatory treatment.

**Main Outcomes Measures:**

All participants were examined preoperatively and postoperatively for ocular symptom score (OSS), noninvasive keratographic tear break-up time (NIKBUT), corneal fluorescein staining, Schirmer I test, lid margin, meibum quality and expressibility, and meibomian gland dropout.

**Results:**

Ocular surface disorders and MGD showed aggravated status at 1 month postoperatively in Cohort I and Cohort III, and the aggravated MGD resolved by 3 months postoperatively. At 1 month postoperatively, Cohort II and Cohort III presented high NIKBUT and low OSS, lid margin, and meibum quality and expressibility (Cohort II vs Cohort I: all* P*<0.001, respectively; Cohort III vs Cohort I:* P*=0.011,* P*=0.024,* P*=0.046,* P*=0.045, and* P*=0.012, respectively). Additionally, Cohort II had better outcomes of lid margin and meibum quality and expressibility than Cohort III at 1 month postoperatively (*P*=0.031,* P*=0.026, and* P*<0.001, respectively). At 3 months postoperatively, Cohort II presented a significantly higher NIKBUT than Cohort I and Cohort III (*P*<0.001 and* P*=0.001, respectively).

**Conclusion:**

Preoperative management of MGD is effective and optimal in alleviating obstructive-MGD and dry eye induced by cataract surgery.

## 1. Introduction

Dry eye, which has been attributed primarily to the tear film dysfunction, is one of the most frequent complaints after cataract surgery; however, the deterioration of the tear film layer after cataract surgery is multifactorial [1–5]. Researchers have found the prevalence of meibomian gland dysfunction (MGD) after cataract surgery and explored the relationship of them; however, the correlation between them was not fully consistent. Han et al. suggested that cataract surgery seems to alter the function of the meibomian glands (MGs) without accompanying structural changes; also the ocular symptom scores remained unrecovered at 3 months postoperatively [5]. However, Kim et al. found that the increased OSDI scores and decreased tear film lipid layer thickness returned to baseline levels at 3 months postoperatively [6]. In addition, Park et al. revealed that MGD was aggravated accompanying MGs structural changes after cataract surgery [7]. Jung et al. suggested that the extent to which the MGD grade was aggravated following cataract surgery differed based on preoperative MGD grade [8]. To date, the exact mechanism by which cataract surgery impairs MGs function remains unknown.

Previous studies reported that patients with obstructive MGD have an abnormal tear film lipid layer thickness, which is responsible for tear film instability [9–12]. Clinically, we found that cataract surgery worsened the lid margin, representing with signs of obstructive MGD (obstructed orifices and thickened lid margin) and inflammation (vascular engorgement). We speculated that inflammation due to cataract surgery might impair MGs function. To date, the role of inflammation in the etiology of obstructive MGD remains equivocal. Matsumoto et al. reported there was a potential presence of periglandular inflammatory cells in patients with obstructive MGD by in vivo confocal microscopy, although the method had a limited power to differentiate clearly between activated stromal cells and leukocytes [13]. Previous studies have suggested that commensal bacteria growing in the MGs can degrade meibomian lipids by lipases and esterases, and the lipid breakdown products, such as free fatty acids, can cause inflammation and hyperkeratinization [14–16]. Additionally, inflammatory cytokines, such as IL-1*α*, can induce keratinization of normal epithelial cells, which results in the obstruction of the sebaceous gland [17]. Several studies focused on elevated levels of tear cytokines in the inferior tear meniscus of patients with MGD [18, 19]; however, the inflammatory status of MGs could not be definitely evaluated by tear cytokines analysis, which is susceptible to postoperative medication interference.

MGD induced by cataract surgery has been proved responsible for the postoperative ocular discomfort and dry eye. However, whether the prophylactic treatment of MGD in cataract patients can alleviate or halt the MGD and dry eye syndrome following cataract surgery has not been investigated. Herein, we attempted to investigate the effect of preoperative treatment and postoperative enhanced anti-inflammatory treatment on alleviating obstructive MGD and dry eye induced by cataract surgery, and to analyze the role of inflammation in the aggravation of obstructive MGD following cataract surgery.

## 2. Methods

### 2.1. Study Design and Inclusion and Exclusion Criteria

This study protocol followed the tenets of the Declaration of Helsinki and was approved by the Institutional Review Board and the Ethics Committee of The Third People's Hospital of Changzhou (No.2018-02). The study trial was registered at http://www.chictr.org.cn (identification No. ChiCTR1800015698). Written informed consent was obtained from all participants in this study; informed consent for the online open access publication of information from participants was also obtained.

We conducted a prospective, randomized clinical trial, and 120 cataract patients (120 eyes) with moderate obstructive MGD were randomized with 2:1:1 into three cohorts according to the random number table by Dr. Peng Song at the beginning of the research (Cohort I, 60 patients (60 eyes); Cohort II, 30 patients (30 eyes); Cohort III, 30 patients (30 eyes)). All participants accepted standardized cataract surgery. Cohort I and the Cohort II were given the routine postoperative administration of anti-inflammatory eye drops, while Cohort III were given the enhanced anti-inflammatory treatment postoperatively. Cohort II accepted preoperative management until the MGD status alleviated significantly (lid marginal abnormality (scores ≤ 1), one or both of meibum quality (scores ≤ 2) and expressibility (scores ≤ 2)), and then underwent cataract surgery.

Eligibility criteria included males and nonpregnant females aged 50-70 years, with a clinical diagnosis of cataract accompanying moderate obstructive MGD. Exclusion criteria included staphylococcal or anterior seborrheic blepharitis, seborrheic MGD, mild or severe obstructive MGD, undergoing lid management, other comorbid ocular diseases except cataract or system diseases (such as diabetes, Sjogren's syndrome, rheumatoid disease, and systemic lupus erythematosus), continuous use of topical ocular medications or systemic drugs (such as NSAIDS, antidepressants, and antihypertensives), and histories of ocular or craniofacial surgery. Patients who already had mild or severe obstructive MGD and worked in high temperature environment or exposed to dust and toxic substances were also excluded.

Obstructive MGD was diagnosed on evidence of plugged MG orifices and hyposecretion of meibum [20]. Eyes were classified and analyzed into none-mild MGD, moderate MGD, and severe MGD according to the preoperative grade of MGD, which was based on three lid parameters [21, 22]: mild MGD, minimally altered lid margin (scores ≤ 1), meibum quality (scores ≤ 2), and expressibility (scores ≤ 2); moderate MGD, moderate altered lid margin (1 < scores ≤ 3), meibum quality (2 < scores ≤ 4), and expressibility (2 < scores ≤ 4); severe MGD, severe altered lid margin ( 3 < scores ≤ 4), meibum quality (4 < scores ≤ 6), and expressibility (4 < scores ≤ 6). Two or more parameters meeting the above criteria are the basis for classification.

### 2.2. Patient Evaluation

All participants were observed and examined preoperatively and at 1 month and 3 months postoperatively for the following: ocular symptom score (OSS), noninvasive keratographic tear break-up time (NIKBUT), corneal fluorescein staining (FL), and Schirmer I test. The parameters of MGs were examined and graded by a slit lamp and Keratograph 5M (OCULUS, Germany), including lid marginal abnormality, meibum quality, meibum expressibility, and MGs dropout. All procedures were performed by the same ophthalmologist. Each patient was told not to apply any eye drops for at least 2 h before each postoperative visit.

The ocular symptom, which included ocular fatigue, discharge, foreign body sensation, dryness, uncomfortable sensation, sticky sensation, pain, epiphora, itchiness, redness, heavy sensation, glare, excessive blinking, and history of chalazion or hordeolum, was evaluated using a questionnaire. The total score of the symptoms ranged from 0 to 14, with higher scores representing a greater severity.

The NIKBUT was calculated during the period in which patients' eyes were continuously open by a Keratograph 5M (OCULUS, Germany). The process was repeated three times for each eye and the average NIKBUT was reported.

The corneal Fl staining was measured for each of the 5 regions of the cornea: central, superior, temporal, nasal, and inferior. The degree of the staining was based on the following: grade 0, no staining; grade 1, superficial stippling and micro-punctate staining; grade 2, macro-punctate staining with some coalescent areas; grade 3, numerous coalescent macro-punctate areas. Each of the 5 regions was graded on a scale from 0 to 3, and the total maximum score was 15 points.

After determining the OSS, NIKBUT, and corneal FL staining, patients were given a 30-minute break and were then given the Schirmer I test. The lid marginal abnormality (lid margin score) was scored from 0 to 4 based on the presence of the following four factors: irregular lid margin, lid engorgement, gland orifice obstruction, and the anterior or posterior displacement of the mucocutaneous junction [23].

The meibum expressibility score of one eye including both the lower and upper eyelid was 0–6. Under the diffusing light of slit lamp, central 5 meibomian glands were observed and squeezed. The activity of MGs (expressibility score) [24] was graded as follows: secretion was seen in all 5 meibomian glands, score 0; 3–4 glands, score1; 1–2 glands, score 2; none of the 5 glands, score 3. The quality of meibum was scored [19] as follows: clear or slight yellow, score 0; creamy yellow, score 1; granular in liquid with white and/or yellow color, score 2; tooth paste shape, score 3. The total meibum score was the sum of the scores of the upper and lower eyelids and was recorded as 0 to 6.

Noncontact Infrared meibography evaluated the MGs dropout using the Keratograph 5M (OCULUS, Germany). Meibomian gland dropout (meibography score) [25] was scored as follows: no absence, score 0; absence less than 1/3 of total glands, score 1; absence more than 1/3 but less than 2/3 of total glands, score 2; absence more than 2/3 of total glands, score 3. The final score of each eye was 0 to 6 points including the upper and lower eyelid.

### 2.3. Cataract Surgery and Drug Regime

All surgeries were performed by the same surgeon (Yajie Sun) under topical anesthesia. No relevant intraoperative complications developed in any case. Participants were directed to apply eye drops correctly and not permitted to wash their faces in the first week postoperatively nor to press or rub the operated eye in the first month postoperatively. No special instructions for lid massage or lid hygiene were given postoperatively. The participants in Cohort II underwent follow-up weekly before cataract surgery, and all participants underwent follow-up biweekly after cataract surgery.

Preoperatively, Cohort II accepted eyelid warm moist compress (moist air and warmer mask, 40°C, 20 minutes) and massage (down or upward mild compression of the eyelids with the finger, 10 minutes), and then topical tobramycin/dexamethasone ointment (tobramycin 0.3% and dexamethasone 0.1%, S.A. ALCON-COUVREUR N.V.) were applied to the lid margin twice per day. The patients in Cohort II were instructed to discontinue instructions 1 week (wash-out period) prior to cataract surgery. Postoperatively, Cohort I and Cohort II accepted tobramycin/dexamethasone eye drops (tobramycin 0.3% and dexamethasone 0.1%, S.A. ALCON-COUVREUR N.V.) 4 times per day in the first week, and the frequency decreased once a week in the following 3 weeks. Cohort III accepted tobramycin/dexamethasone eye drops 6 times per day in the first week and 4 times per day in the second week and the frequency decreased by half every week in the following 2 weeks. All cohorts received 0.1% sodium hyaluronate (Santen Pharmaceutical Co., Ltd.) 4 times per day for the first month postoperatively.

### 2.4. Statistical Analysis

Sample size calculations were based on showing differences of four main parameters (OSS, NIKBUT, lid margin, and meibum quality) through multiple comparisons of two treatments vs a control-simulation. A priori power analysis showed that the maximum sample size required for 80% power of detecting those differences as significant at the two-side 5% level was 21 subjects using the PASS 2008 calculation software. We enrolled 27 eyes and found the power of our study was 90.3%. All statistical analyses were performed using SPSS V.19.0 software. A linear mixed model with Bonferroni post hoc analysis was used to evaluate repeated measurements of continuous variables, including OSS, NIKBUT, and Schirmer I test score. Generalized linear mixed model analysis with Bonferroni post hoc analysis was used for repeated measurements of discrete variables, including the FL score, lid margin findings, and MGs findings. The Student t-test and Mann–Whitney U test were used to compare differences in outcomes between every two cohorts. Chi-square test was used to analyze the enumeration data. Spearman's correlation analysis was used to estimate the correlations between various factors. All statistical analyses were performed using SPSS V.19.0 software.* P* values less than 0.05 were considered statistically significant.

## 3. Results

**Figure 1:**
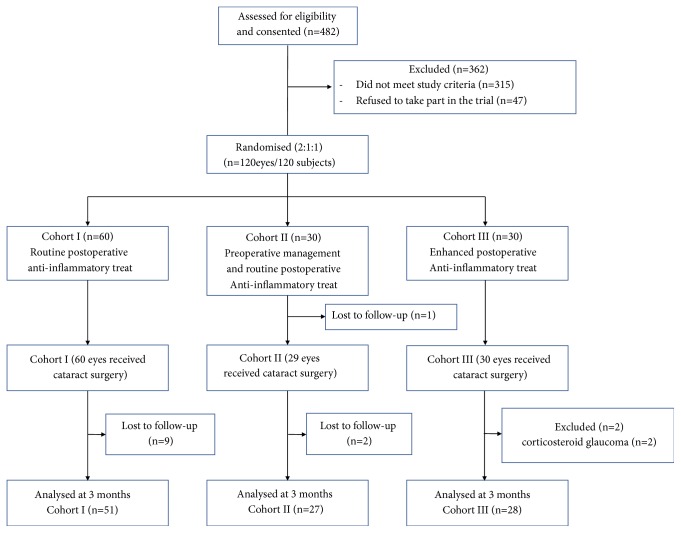
Study design flow diagram.

*Demographics.* Of the 120 patients (120 eyes) enrolled in the study, 12 patients (12 eyes) were lost to follow-up: 9 eyes in Cohort I and 3 eyes in Cohort II. Two patients (2 eyes) in Cohort III suffered corticosteroid glaucoma and were consequently excluded from the study. The remaining 106 patients (106 eyes) participated in the study (Figure 1). The mean age of the 106 patients was 63.19 ± 4.99 years, and there were 50 females. The mean ages of three cohorts were 63.76 ± 4.48 (Cohort I), 62.41 ± 5.81 (Cohort II), and 62.89 ± 5.08 years (Cohort III), respectively. The female proportions of each cohort were 47.1%, 55.6%, and 39.3%, respectively. No significant differences in age and gender were found among the three cohorts (*P *= 0.491 and* P *= 0.485, respectively). The mean time of preoperative treatment in Cohort II was 42.85 ± 8.47 days.

### 3.1. Ocular Surface Disorders and Meibomian Gland Dysfunction in All Cohorts

The ocular surface and MGs parameters are shown in Table 1 (Cohort I), Table 2 (Cohort II), and Table 3 (Cohort III).

In Cohort I, the scores of OSS, corneal FL, lid marginal abnormality, meibum quality, and expressibility were significantly higher than the baselines at 1 month postoperatively (all* P *< 0.001, respectively; Table 1), and these parameters returned to the preoperative level at 3 months postoperatively (*P *= 0.077,* P *= 0.163,* P *= 0.099,* P *= 0.592 and* P *= 0.304, respectively; Table 1). The NIKBUT was significantly decreased at 1 month and 3 months postoperatively (*P *< 0.001 and* P *< 0.001, respectively; Table 1). Lid engorgement was observed in 29 eyes (56.9%) at baseline or preoperation, 44 eyes (86.3%) at 1 month postoperatively, and 34 eyes (66.7%) at 3 months postoperatively (*P *= 0.001 and* P *= 0.308, when compared with baseline, respectively).

In Cohort II, the NIKBUT increased significantly (*P* < 0.001, Table 2) and the scores of OSS, lid marginal abnormality, meibum quality, and expressibility decreased significantly (*P* < 0.001,* P* < 0.001,* P* < 0.001 and* P* < 0.001, respectively, Table 2) at preoperation compared to those at baseline after preoperative treatment. At 1 month postoperatively, NIKBUT decreased significantly when compared with baseline (*P* = 0.003, Table 2) and returned to baseline level at 3 months postoperatively (*P* = 0.989, Table 2). Other parameters were not significantly different when compared with the baselines at 1 month or 3 months postoperatively. Lid engorgement was observed in 15 eyes (55.6%) at baseline, 5 eyes (18.5%) at preoperation, 17 eyes (63.0%) at 1 month postoperatively, and 16 eyes (55.6%) at 3 months postoperatively (*P *= 0.023,* P *= 0.782 and* P *> 0.999, when compared with baseline, respectively).

In Cohort III, the OSS and meibum expressibility score were significantly higher at 1 month postoperatively than they were preoperatively (*P* < 0.001, and* P* = 0.031, respectively; Table 3), and these parameters returned to the preoperative level at 3 months postoperatively (*P* > 0.999 and* P* > 0.999, respectively; Table 3). The NIKBUT decreased significantly at 1 month postoperatively (*P* < 0.001), and it returned to the preoperative level at 3 months postoperatively (*P *= 0.101). Lid engorgement was observed in 17 eyes (60.1%) at baseline or preoperation, 18 eyes (64.3%) at 1 month postoperatively, and 18 eyes (64.3%) at 3 months postoperatively (*P *> 0.999 and* P *> 0.999, when compared with baseline, respectively).

### 3.2. Comparison of Ocular Surface and MGs Parameters among the Three Cohorts

**Figure 2:**
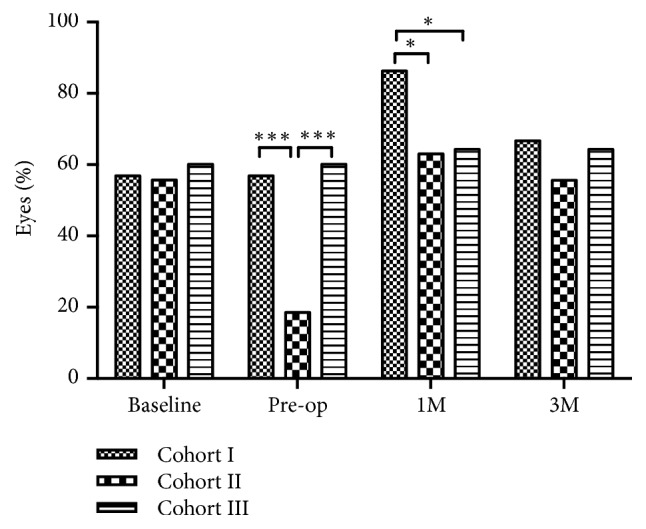
*The rates of lid engorgement in all cohorts* (*∗∗∗P *< 0.001, *∗∗P *< 0.01, *∗P *< 0.05).

**Figure 3:**
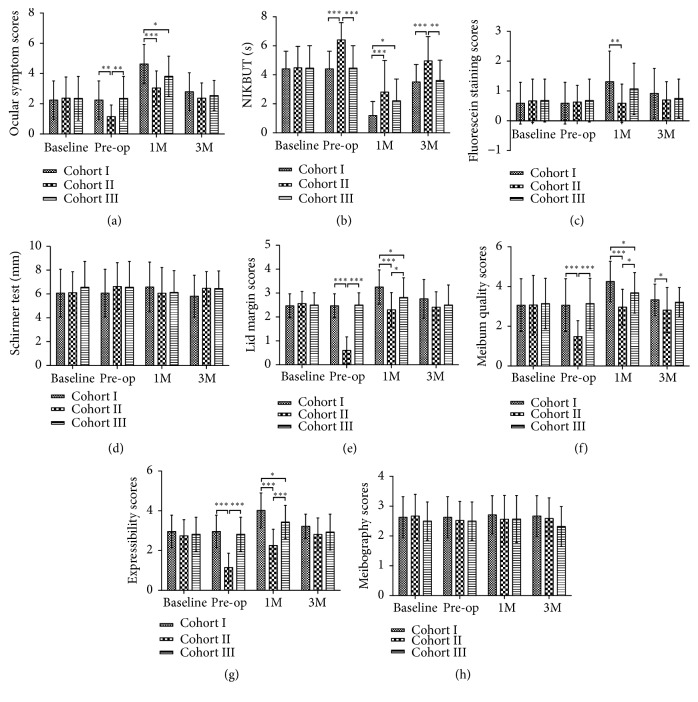
*Comparison of ocular surface and MGs parameters within the three cohorts at 3 investigated points (baseline, pre-operation, and 1 month and 3 months postoperatively).* (a) Ocular symptom. (b) NIKBUT. (c) Corneal fluorescein staining. (d) Schirmer test. (e) Lid margin. (f) Meibum quality. (g) Meibum expressibility. (h) Meibography (MG dropout) (*∗∗∗P *< 0.001, *∗∗P *< 0.01, *∗P *< 0.05).

A detailed comparison of the ocular surface and MGs parameters for the three cohorts is shown in Figure 3. Comparisons of the rates of lid engorgement among the three cohorts are shown in Figure 2.

At baseline, there was no statistical difference found of all parameters among three cohorts. After giving preoperative management of MGD, Cohort II showed a significantly lower score of OSS and a longer NIKBUT than Cohort I (*P* = 0.001 and* P* < 0.001, respectively; Figures 3(a) and 3(b)) and Cohort III (*P* = 0.004 and* P* < 0.001, respectively; Figures 3(a) and 3(b)). This indicated that there was better tear film stability in Cohort II. The MG parameters, which included scores of lid margin, meibum quality, and expressibility, were significantly lower in Cohort II than in Cohort I (all* P* < 0.001, respectively; Figures 3(e), 3(f), and 3(g)) and Cohort III (all* P* < 0.001, respectively, Figures 3(e), 3(f), and 3(g)), which showed a conspicuous alleviation of MGD after the preoperative treatment. However, the parameters of FL score, Schirmer I test, and meibography score did not show differences among the three cohorts at preoperation (*P* = 0.849,* P* = 0.424 and* P* = 0.656, respectively, Figures 3(c), 3(d), and 3(h)). In addition, Cohort II had lower rates of lid engorgement than Cohort I and Cohort III at preoperation (*P* =0.001 and* P* = 0.003, respectively, Figure 2).

At 1 month postoperatively, the majority of the ocular surface parameters (OSS, NIKBUT, and FL) and the MGs parameters (lid margin, meibum quality, and expressibility) in Cohort II were significantly superior to the parameters in Cohort I (*P* < 0.001,* P* < 0.001,* P* = 0.003,* P* < 0.001,* P* < 0.001 and* P* < 0.001, respectively; Figures 3(a), 3(b), 3(c), 3(e), 3(f), and 3(g)). Cohort III had a significantly higher NIKBUT (*P* = 0.011, Figure 3(b)) and lower scores of ocular symptoms, lid margin, meibum quality, and expressibility than Cohort I (*P* = 0.024,* P* = 0.046,* P* = 0.045, and* P* = 0.012, respectively; Figures 3(a), 3(e), 3(f), and 3(g)). In addition, Cohort II had better results of lid margin, meibum quality, and expressibility than Cohort III (*P* = 0.031,* P* = 0.026 and* P* < 0.001, respectively; Figures 3(e), 3(f), and 3(g)). The rate of engorgement in Cohort I was higher than that in Cohort II and Cohort III (*P* = 0.018 and* P* = 0.024, respectively, Figure 2).

At 3 months postoperatively, Cohort II presented a significantly higher NIKBUT than Cohort I and Cohort III (*P*<0.001 and* P*=0.001, respectively; Figure 3(b)). Additionally, meibum quality in Cohort II was significantly better than that in Cohort I at 3 months postoperatively (*P* = 0.045, Figure 3(f)). These data suggest that the management of MGD before cataract surgery may result in better outcomes than those with or without enhanced anti-inflammatory treatment postoperatively.

### 3.3. Analysis of the Correlations between the Changes of MGD Parameters and OSS or NIKBUT in Cohort I at 1 Month Postoperatively

**Figure 4:**
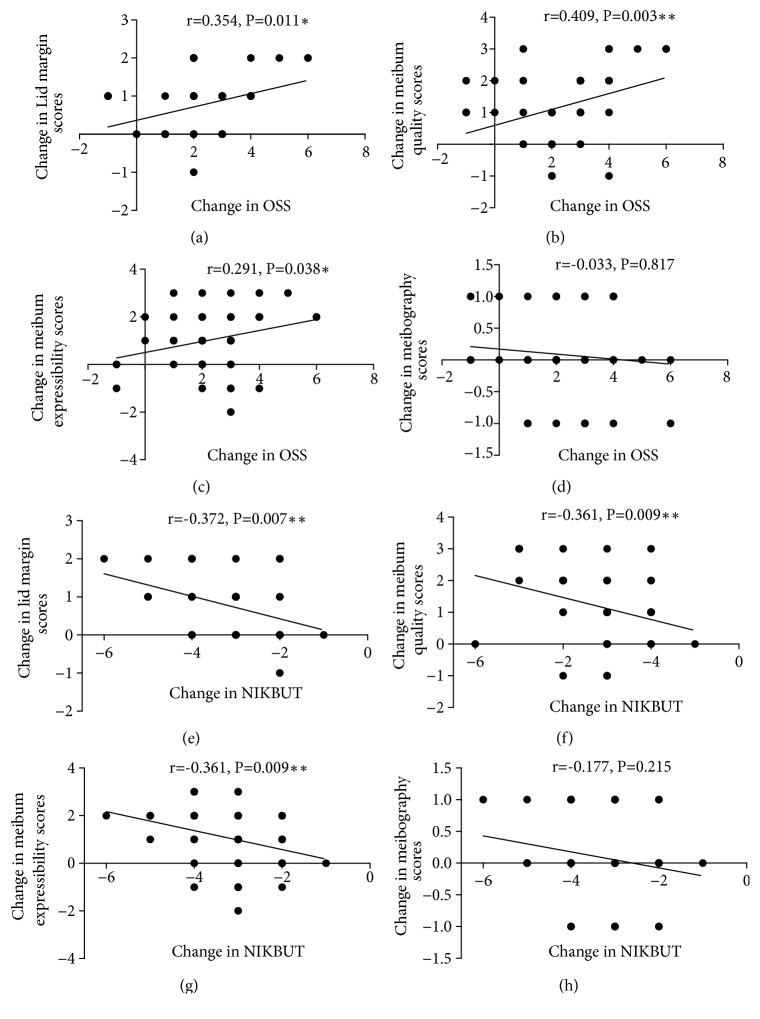
*Analysis of the correlations between the parametric changes in MGD and changes in OSS or NIKBUT in Cohort I at one month postoperatively.* ((a)-(d)) The correlation between the change in OSS and changes in lid margin score, meibum quality score, meibum expressibility score, and meibography score, respectively. ((e)-(h)) The correlation between the change in NIKBUT with changes in lid margin score, meibum quality score, meibum expressibility score, and meibography score, respectively (*∗∗∗P *< 0.001, *∗∗P *< 0.01, *∗P *< 0.05).

The changes in scores of lid margin, meibum quality, and expressibility at 1 month postoperatively were significantly and positively correlated with the change in OSS (r = 0.354,* P* = 0.011, Figure 4(a); r = 0.409,* P* = 0.003, Figure 4(b); r = 0.291,* P* = 0.038, Figure 4(c); respectively). However, the change in OSS showed no significant correlation with change in meibography score (r = -0.033,* P* = 0.817, Figure 4(d)). The change in NIKBUT showed significant negative correlation with changes in scores of lid margin, meibum quality, and expressibility (r = -0.372,* P* = 0.007, Figure 4(e); r = -0.361,* P* = 0.009, Figure 4(f); r = -0.352,* P* = 0.011, Figure 4(g); respectively). In addition, the change in NIKBUT showed no significant correlation with change in meibography score (r = -0.177,* P* = 0.215, Figure 4(h)).

## 4. Discussion

The prevalence of dry eye symptoms after cataract surgery highlights the need to investigate how cataract surgery impairs MGs function and homeostasis of ocular surface, and to seek effective measures to alleviate the impairment. In the present study, the development and aggravation of obstructive MGD and tear film dyshomeostasis were revealed at 1 month postoperatively, and the MGs parameters returned to the preoperative level by 3 months postoperatively in all cohorts, which indicated that cataract surgery may not aggravate moderate obstructive MGD under routine postoperative management in long-term follow-up. The most notable finding was that the preoperative management and postoperative enhanced anti-inflammatory treatment can lead to significantly better outcomes of ocular surface and MGs than routine postoperative treatment.

Several studies have reported that cataract surgery can lead to the development or aggravation of dry eye and MGD, as represented by ocular surface and MGs parameter changes [3, 5, 6], although the details of these changes were inconsistent. Consistent with these reports, we discovered that there were significant changes in OSS, NIKBUT, lid margin, meibum quality, and expressibility in moderate MGD patients after cataract surgery. In addition, we did not find significant changes in the tear secretion and MGs dropout after cataract surgery. In the present study, Spearman's correlation analysis revealed that changes in OSS and NIKBUT in moderate MGD patients were correlated with the changes in some MGD parameters at 1 month postoperatively. We suggest that tear film instability and evaporative dry eye after cataract surgery can be attributed to the aggravation of obstructive MGD without a decrease in aqueous tear production. In addition, the MGs parameters in Cohort I and Cohort III were found to have returned to the preoperative level at 3 months postoperatively, which indicated that cataract surgery may not aggravate moderate obstructive MGD in long-term follow-up. In Cohort I, although the NIKBUT at 3 months significantly increased compared to that at 1 month postoperatively, it was still shorter than the preoperative level. It has been demonstrated that the functional change of meibum, which is due to the alteration of tear film lipids and lipid-protein interactions, contributes to tear film instability and the aggravation of obstruction in MGs [10]. We suspected that tear film homeostasis in Cohort I remains disturbed at 3 months postoperatively, and it may require longer time to revert to the preoperative status.

In the present study, preoperative management and enhanced postoperative anti-inflammatory treatment significantly decreased the rate of lid engorgement at 1 month postoperatively. Lid vascular engorgement is an important marker of ocular inflammation, and it has greater stability than tear cytokines test when investigating MGD, because it avoids the instantaneous effect of postoperative medication on the tear sample analysis. We speculated that anti-inflammatory treatment may be responsible for the mechanism to alleviate the aggravation of the MGD.

Accumulating evidence implies that inflammatory factors may be involved in the process of obstructive MGD. Obstructive MGD is commonly characterized by terminal duct obstruction with thickened opaque meibum containing keratinized cell materials [21]. The classic pathophysiological mechanisms of obstructive MGD are hyperkeratinization and acinar atrophy, which are attributed to increasing intraglandular pressure and aberrant differentiation or migration of meibomian gland stem cells [26]. Researchers have found that, under increasing intraglandular pressure and lipid peroxidation, activated epithelial cells, even normal and stressed sebocytes, can produce inflammatory cytokines such as TNF and interleukin, which promote a subclinical inflammatory microenvironment [27–29]. In turn, inflammatory cytokines also activate epithelial cells and induce increased keratinization [17]. Taken together, a vicious circle [30] formed and the subclinical inflammation may play an important role in the development of obstructive MGD. Giant papillary conjunctivitis induced by CL wear promoted the development of obstructive MGD, as suggested by Mathers and Billborough [31], which may support the hypothesis of the potential involvement of inflammatory mediators in the pathogenesis of obstructive MGD. We speculated that inflammatory mediators which come from the onset of postoperative inflammatory cascades find their way from the ocular surface through the tarsus toward the acinus and ductules and amplify the subclinical inflammation in MGs, as well as the downstream events, such as changes in meibum composition, hyperkeratinization, and obstruction of the MGs.

The most outstanding finding was that the preoperative management of MGD can lead to significantly better outcomes of ocular surface and MGs, which suggested preoperative management may be an effective measure to alleviate the ocular discomfort and dry eye induced by cataract surgery. The warm moist compress and massage could promote the delivery of MG secretions [32], so as to discharge abnormal meibum and reduce intraglandular pressure. Studies have shown that warm moist compress could alter MG secretions and increase the tear film lipid layer thickness, and these were found to be significantly related to the reduction of symptom scores [33, 34]. We have no idea whether the warm moist compress and massage could play an anti-inflammatory role. Actually, these can reduce the intraglandular pressure and may decrease the stress on meibocytes and activation of inflammation. To further understand the role of inflammation in the aggravation of obstructive MGD induced by cataract surgery, we performed enhanced postoperative anti-inflammatory treatment. The result showed significantly better outcomes of ocular surface and MGs in Cohort III than in Cohort I at 1 month postoperatively. We suggested that preoperative management of MGD may attenuate the level of subclinical inflammatory microenvironment, and postoperative enhanced anti-inflammatory treatment can inhibit the instantaneous inflammation induced by cataract surgery.

In this study, the preoperative management had better outcomes of ocular surface and MGs than enhanced postoperative anti-inflammatory treatment at 1 month postoperatively. In addition, 2 patients in Cohort III suffered corticosteroid glaucoma, suggesting that enhanced usage of corticosteroids eye drops could raise the risk of corticosteroid glaucoma. These results suggested that the preoperative evaluation and management of MGD are important and the optimal choice.

The current study found preoperative management and postoperative enhanced anti-inflammatory treatment would have better effects on ocular surface and MGs than routine postoperative treatment. However, there are several drawbacks that should be noticed. Firstly, the study did not have placebo treatment for preoperative management of MGD in Cohort II and Cohort III. Secondly, patients must receive postoperative anti-inflammatory drugs and are forbidden from pressing eyeball and massaging eyelids after cataract surgery, because of the leaving none-sutured corneal incision by phacoemulsification. Considering patients' safety, we did not design a control cohort without postoperative anti-inflammatory treatment, nor did we design a cohort with warm moist compress and massage after cataract surgery. These may lead to bias in the analysis of the role of inflammation in MGD induced by cataract surgery or in the analysis of differences between preoperative and postoperative treatment.

In summary, our data revealed that obstructive MGD may be aggravated by cataract surgery in the short term, and the aggravated status recovered at 3 months postoperatively. More significantly, anti-inflammatory management is effective in alleviating MGD and dry eye induced by cataract surgery. The preoperative management had significantly better MGs outcomes than postoperative enhanced anti-inflammatory treatment. All in all, our study advocated that the preoperative evaluation and management could alleviate the impairment of MGs function induced by cataract surgery and implied that inflammation may play an important role in the process of aggravation of obstructive MGD after cataract surgery.

## Figures and Tables

**Table 1:** Outcomes of ocular surface and MGs parameters in Cohort I.

Parameters	Mean±SD			*P*-value		
	Baseline/Pre-op^c^	1 month	3 months	Overrall	1M VS	3M VS
					Baseline	Baseline
OSS^a^	2.24±1.27	4.63±1.30	2.80±1.25	<0.001*∗∗∗*	<0.001*∗∗∗*	0.077

NIKBUT(s)^a^	4.41±1.22	1.20±0.96	3.51±1.21	<0.001*∗∗∗*	<0.001*∗∗∗*	<0.001*∗∗∗*

Corneal FL^b^	0.59±0.70	1.31±1.03	0.92±0.84	<0.001*∗∗∗*	<0.001*∗∗∗*	0.163

Schirmer(mm)^a^	6.08±2.00	6.59±2.09	5.82±1.76	0.136	0.570	>0.999

Lid margin^b^	2.47±0.50	3.25±0.72	2.76±0.81	<0.001*∗∗∗*	<0.001*∗∗∗*	0.099

Meibum quality^b^	3.06±1.33	4.25±1.02	3.33±0.79	<0.001*∗∗∗*	<0.001*∗∗∗*	0.592

Expressibility^b^	2.96±0.82	4.02±0.88	3.22±0.61	<0.001*∗∗∗*	<0.001*∗∗∗*	0.304

Meibography^b^	2.63±0.70	2.71±0.64	2.67±0.68	0.841	>0.999	>0.999

^a^Continuous values were analyzed by linear mixed model with Bonferroni post hoc analysis. ^b^Noncontinuous values were analyzed by generalized linear mixed model analysis with Bonferroni post hoc analysis. ^c^The investigative point of preoperation in Cohort I was the same point as the baseline (*∗∗∗P *< 0.001, *∗∗P *< 0.01, *∗P *< 0.05).

**Table 2:** Outcomes of ocular surface and MGs parameters in Cohort II.

Parameters	Mean±SD				*P*-value			
	Baseline	Pre-op	1 month	3 months	Overrall^c^	Pre-op VS	1M VS	3M VS
						Baseline	Baseline	Baseline
OSS^a^	2.37±1.40	1.15±0.77	3.04±1.13	2.37±1.01	0.052	<0.001*∗∗∗*	0.164	>0.999

NIKBUT(s)^a^	4.48±1.48	6.41±1.19	2.81±2.18	4.96±1.68	<0.001*∗∗∗*	<0.001*∗∗∗*	0.003*∗∗*	0.989

Corneal FL^b^	0.67±0.73	0.63±0.56	0.59±0.64	0.70±0.61	0.821	0.836	>0.999	>0.999

Schirmer(mm)^a^	6.11±1.76	6.63±2.00	6.07±2.15	6.48±1.40	0.655	0.317	>0.999	>0.999

Lid margin^b^	2.56±0.51	0.59±0.57	2.30±0.72	2.41±0.64	0.320	<0.001*∗∗∗*	0.401	>0.999

Meibum quality^b^	3.07±1.49	1.48±0.80	2.96±0.90	2.81±1.14	0.730	<0.001*∗∗∗*	>0.999	>0.999

Expressibility^b^	2.74±0.81	1.15±0.72	2.26±0.81	2.81±0.83	0.030	<0.001*∗∗∗*	0.102	>0.999

Meibography^b^	2.67±0.73	2.52±0.64	2.56±0.80	2.59±0.69	0.856	0.434	>0.999	>0.999

^a^Continuous values were analyzed by linear mixed model with Bonferroni post hoc analysis. ^b^Noncontinuous values were analyzed by generalized linear mixed model analysis with Bonferroni post hoc analysis. ^c^The overall analysis included baseline and 1 month and 3 months postoperatively (*∗∗∗P *< 0.001, *∗∗P *< 0.01, *∗P *< 0.05).

**Table 3:** Outcomes of ocular surface and MGs parameters in Cohort III.

Parameters	Mean±SD			*P*-value		
	Baseline/Pre-op^c^	1 month	3 months	Overrall	1M VS	3M VS
					Baseline	Baseline
OSS^**a**^	2.36±1.45	3.82±1.33	2.54±1.00	<0.001*∗∗∗*	<0.001*∗∗∗*	>0.999

NIKBUT(s)^a^	4.46±1.55	2.21±1.50	3.61±1.40	<0.001*∗∗∗*	<0.001*∗∗∗*	0.101

Corneal FL^b^	0.68±0.72	1.07±0.86	0.75±0.65	0.118	0.158	>0.999

Schirmer(mm)^a^	6.57±2.17	6.14±1.82	6.46±1.48	0.665	>0.999	>0.999

Lid margin	2.50±0.51	2.82±0.82	2.50±0.84	0.177	0.321	>0.999

Meibum quality^b^	3.14±1.27	3.68±1.02	3.21±0.74	0.115	0.167	>0.999

Expressibility^b^	2.82±0.86	3.43±0.84	2.93±0.90	0.024*∗*	0.031*∗*	>0.999

Meibography^b^	2.50±0.64	2.57±0.80	2.32±0.67	0.394	>0.999	>0.999

^a^Continuous values were analyzed by linear mixed model with Bonferroni post hoc analysis. ^b^Noncontinuous values were analyzed by generalized linear mixed model analysis with Bonferroni post hoc analysis. ^c^The investigative point of preoperation in Cohort III was the same point as the baseline (*∗∗∗P *< 0.001, *∗∗P *< 0.01, *∗P *< 0.05).

## Data Availability

The datasets used and/or analyzed during the present study are available from the corresponding author on reasonable request.
